# Phentermine Use During First and Second Trimesters Associated with Fetal Stroke

**DOI:** 10.7759/cureus.6170

**Published:** 2019-11-16

**Authors:** Nathan D'Adesky, Suman Ghosh

**Affiliations:** 1 Neurology, University of Florida College of Medicine, Gainesville, USA; 2 Pediatric Neurology, University of Florida College of Medicine, Gainesville, USA

**Keywords:** fetal, stroke, in-utero, phentermine, porencephaly, weight loss, sympathomimetic, weight loss medications, fetus, pregnancy

## Abstract

Phentermine is a sympathomimetic amine used for the short-term weight loss that has been associated with ischemic and hemorrhagic strokes in adults. The effects of this medication on a developing fetus are not well studied. We present the case of a woman who was taking phentermine during the first two trimesters of pregnancy and subsequently delivered a child with bilateral porencephalic cysts likely due to a prenatal stroke.

## Introduction

Phentermine is a commonly prescribed, Food and Drug Administration (FDA) approved, appetite suppressant medication in the United States for weight loss with short-term use (<12 weeks) as an adjunct to lifestyle modifications such as diet and exercise. It is a sympathomimetic amine which increases dopamine, norepinephrine, and serotonin levels [1,2]. Drugs like phentermine used to aid in weight loss are predominantly used by females 17-64 years of age, a population with childbearing potential [2,3]. Weight loss and the use of weight loss supplements are contraindicated during pregnancy. It is not uncommon to be unaware of pregnancy up to the first trimester, and factors such as obesity can contribute to a delay in the awareness of pregnancy [4]. The effects of weight loss supplements on fetal growth and development are not well known [5].

Despite proven efficacy of appetite suppressants in weight reduction, there are still concerns about adverse side effects. Phentermine is one of the few sympathomimetic appetite suppressants that is left on the market after several drugs of the same class were discontinued due to adverse cardiovascular outcomes. Fenfluramine and dexfenfluramine have been associated with valvular heart disease and pulmonary hypertension, aminorex for pulmonary hypertension, and sibutramine for cardiovascular disease [6-8]. Although phentermine is still on the market, it has been associated with stroke, hypertension, palpitations, and tachycardia. Studies have shown that combination drugs containing phentermine have been associated with valvular dysfunction and pulmonary hypertension [8-10]. This case report demonstrates the possible association of fetal stroke in the setting of maternal phentermine use during the first and second trimesters of pregnancy.

## Case presentation

**Figure 1:**
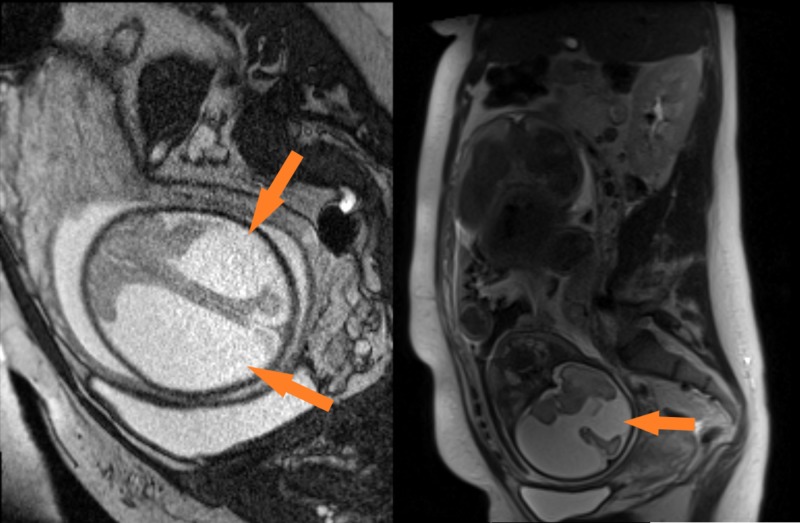
Fetal MRI at 28 weeks Images from fetal MRI at 28 weeks showing porencephaly in bilateral middle cerebral artery territories.

**Figure 2:**
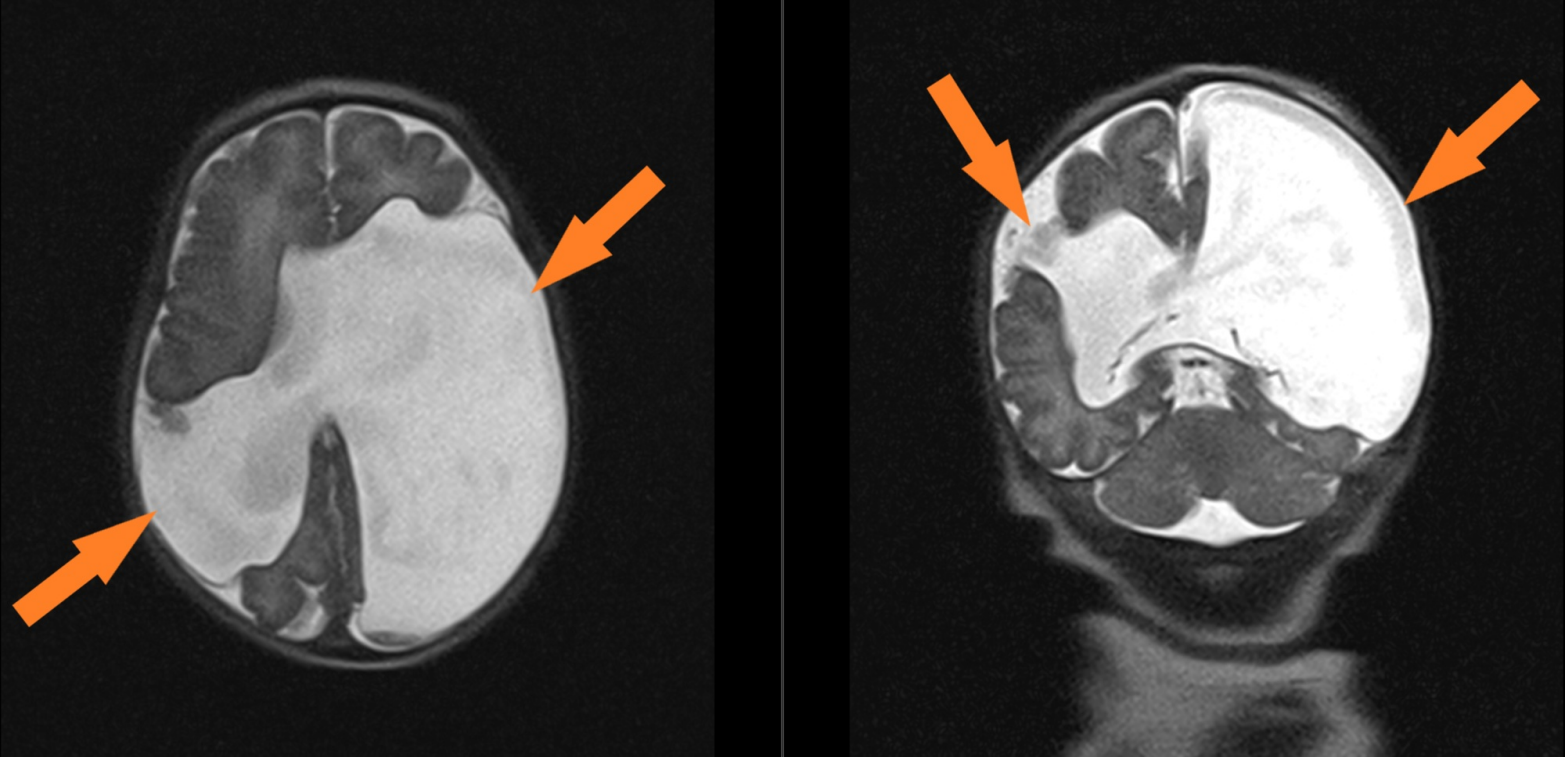
Neonatal MRI at day of life 1 Images from patient’s brain MRI at day of life 1 showing porencephaly in the bilateral middle cerebral artery territories.

A baby girl with bilateral porencephalic cysts was born at 40-week gestation via vaginal birth after caesarian section delivery to a 24-year-old gravida 4 para 4 mother with a history of anemia, prior caesarian section in 2015, and a body mass index of 27. The mother had no past medical history of hypertension, diabetes, deep vein thrombosis, cardiovascular disease, sickle cell disease, or sexually transmitted diseases. The mother denied the use of alcohol, tobacco, or illicit drug use during or before pregnancy, but she did admitted to the use of phentermine while she was pregnant. She reported that she was not aware of her pregnancy until she was at approximately 26-28 weeks of gestation after receiving a urine pregnancy test during a routine medical visit. At that time, she discontinued her phentermine. A urine pregnancy test was not conducted prior to starting phentermine. She was on no other medications prior to or during the pregnancy. All prenatal labs were normal with normal serology. She did not have gestational diabetes, pregnancy-related hypertension, maternal infections, or other maternal complications during the pregnancy. Brain abnormalities were first seen during prenatal ultrasound at 28 weeks of gestation, which demonstrated bilateral enlarged ventricles. Fetal magnetic resonance imaging (MRI) was performed to confirm the findings (Figure 1). Delivery was uncomplicated with Apgar scores of 8 and 9 at 1 and 5 minute(s), respectively. Birth weight was 3.37 kilograms, length was 50 centimeters (cm) and head circumference was 36 cm. The patient’s physical examination was significant for increased tone and brisk reflexes in her upper and lower extremities bilaterally. Patient was admitted to the Neonatal Intensive Care Unit for observation and discharged after three days in the hospital. MRI brain at day 1 of life revealed bilateral porencephalic cysts with thinning of the right cerebral cortex and absence of a significant region of the left cerebral cortex. Occipital lobes were dysplastic bilaterally (Figure 2). These findings suggest an event of bilateral middle cerebral artery (MCA) stroke in the left MCA and posterior right MCA. The infant went on to develop spastic quadriplegic cerebral palsy and global developmental delay

## Discussion

In this case study, the patient suffered from an in utero stroke resulting in cerebral palsy and global developmental delay. Ischemic or hemorrhagic injury to the brain before birth most likely resulted in porencephalic cysts. Porencephaly can be caused by events such as an ischemic infarct or a prenatal infection causing tissue necrosis. Risk factors that contribute to porencephaly include monozygotic twins, infection, prothrombotic conditions, maternal cardiac arrest, or sympathomimetics [11].

There is limited data on the potential fetal risks involved in the use of sympathomimetic appetite suppressants during pregnancy, specifically with the use of phentermine alone. Prospective cohort studies showed no difference in pregnancy loss or major structural anomalies in infants exposed to phentermine, fenfluramine, dexfenfluramine, or combined phentermine/fenfluramine [12,13]. There is significant data involving sympathomimetic appetite suppressants on cardiovascular disease including stroke, mitral valvular disease, and pulmonary hypertension with both ischemic and hemorrhagic strokes being associated with phentermine use [6,10,14]. Caution should be taken in prescribing this medication during pregnancy regardless due to the contraindication of weight loss during pregnancy.

## Conclusions

In this case presentation, a woman who was taking phentermine during the first two trimesters of pregnancy delivered a child who suffered from a large lesion likely due to a prenatal stroke. It is still uncertain that phentermine was the cause of the stroke; however, given the lack of maternal/fetal risk factors, the effects of phentermine during the first two trimesters should not be overlooked.
